# A Physics-Based Compact Model for P-Type Ballistic Nanowire GAA MOSFETs Incorporating the Source-to-Drain Tunneling Effect

**DOI:** 10.3390/nano16171053

**Published:** 2026-08-24

**Authors:** He Cheng, Zhijia Yang, Chao Zhang, Zhipeng Zhang

**Affiliations:** 1 State Key Laboratory of Robotics and Intelligent Systems, Shenyang Institute of Automation, Chinese Academy of Sciences, Shenyang 110016, China; chenghe@sia.cn; 2 Shenyang Institute of Automation, Chinese Academy of Sciences, Shenyang 110016, China; yang@sia.cn (Z.Y.); zhangchao@sia.cn (C.Z.)

**Keywords:** ballistic transport, compact model, sub-7 nm p-type nanowire GAA MOSFET, source-to-drain tunneling, WKB approximation, Verilog-A implementation

## Abstract

This paper presents an analytical compact DC current model and a numerical gate capacitance model for p-type cylindrical gate-all-around (GAA) nanowire metal–oxide–semiconductor field-effect transistors (MOSFETs). The models are formulated within the Landauer transport framework, incorporating source-to-drain tunneling (SDT) and quantum statistical charge analysis. The proposed current model is validated against non-equilibrium Green’s function (NEGF) simulations for different channel lengths, nanowire radii, and bias conditions, showing good agreement with the NEGF results in the ballistic limit. The model parameters are separated into physical parameters obtained or calibrated from the NEGF simulations and a single set of global empirical fitting parameters. The latter is extracted once and remains unchanged across the investigated device geometries and bias conditions, allowing its transferability to be evaluated. The compact model is implemented in Verilog-A, and its SPICE compatibility is verified through DC simulations of PMOS inverter circuits. All NEGF comparisons in this work are performed with a zero channel backscattering coefficient corresponding to the ballistic transport limit; validation of the quasi-ballistic regime is left for future work.

## 1. Introduction

According to the International Roadmap for Devices and Systems (IRDS), both the gate length and the silicon channel thickness are expected to enter the sub-7 nm regime [1]. As metal–oxide–semiconductor field-effect transistor (MOSFET) dimensions continue to scale below 7 nm, increasingly stringent electrostatic gate control is required to suppress short-channel effects (SCEs), including threshold voltage roll-off and drain-induced barrier lowering (DIBL) [2,3,4,5]. Owing to their superior electrostatic integrity and excellent immunity to SCEs, gate-all-around (GAA) MOSFETs have become one of the most promising device architectures for future complementary metal–oxide–semiconductor (CMOS) technologies. To further improve integration density and drive capability, multi-stacked silicon nanosheet GAA structures have been proposed to increase the effective channel width [6,7,8,9,10,11,12]. More recently, complementary field-effect transistors (CFETs) based on two-dimensional semiconductors with a contacted poly pitch of 50 nm have been experimentally demonstrated, further highlighting the scalability and application potential of GAA-based device architectures for future logic technologies [13]. Although nanosheet GAA transistors have already entered commercial production [14,15], cylindrical nanowire GAA MOSFETs continue to provide an attractive platform for analytical modeling of deeply scaled devices. Their highly symmetric geometry permits closed-form electrostatic solutions, making them particularly suitable for investigating quantum confinement and ballistic transport effects while facilitating the development of physics-based compact models. Furthermore, the resulting modeling methodology can be naturally extended to nanosheet GAA FETs and CFETs through appropriate formulations of subband structures and density of states (DOS) [9,16,17]. Beyond advanced logic applications, GAA MOSFETs have also demonstrated significant potential in emerging fields such as radiation-hardened integrated circuits and gas sensors [18,19].

As the channel length approaches the carrier mean free path in silicon [20], carrier transport gradually transitions from the diffusive regime to the quasi-ballistic and ballistic regimes. In this regime, quantum confinement and phonon scattering exert a significant influence on carrier transport [15,21]. Consequently, accurate modeling of deeply scaled devices requires the simultaneous consideration of quantum confinement and ballistic transport effects. As device dimensions continue to shrink into the sub-7 nm regime, source-to-drain tunneling (SDT) becomes increasingly significant, particularly in the subthreshold region. Furthermore, the strong anisotropy of the valence band results in pronounced orientation-dependent hole transport in p-type devices, leading to substantial variations in their electrical characteristics. Therefore, there remains a strong need for an analytical compact model that incorporates SDT while maintaining accuracy over the entire operating regime. Although advanced numerical approaches, including the non-equilibrium Green’s function (NEGF) formalism, *ab initio* simulations, and the k·p method, can accurately describe quantum and ballistic transport phenomena [11,15,20,21,22], their high computational complexity makes them impractical for circuit-level simulations. To address this issue, various analytical compact models have been developed for ballistic GAA MOSFETs [23,24,25,26,27], including models that account for thermionic emission and SDT [28,29]. More recently, analytical compact models for short-channel p-type GAA MOSFETs have also been reported. In particular, our previous work proposed a DIBL-aware analytical compact model for short-channel p-type GAA MOSFETs [30]. However, that model neglected the SDT effect, limiting its applicability to aggressively scaled devices where tunneling plays an increasingly important role.

In this work, we develop an analytical compact model for ultra-short-channel p-type cylindrical GAA MOSFETs that includes ballistic transport, quantum confinement, DIBL, and SDT. The formulation retains the channel backscattering coefficient $R_{\mathrm{ref}}$ in the Landauer transmission expression, allowing the same framework to be extended to quasi-ballistic transport; all results reported in this work, however, correspond to $R_{\mathrm{ref}}=0$. A numerical gate capacitance model is also constructed based on the SDT-influenced channel hole charge density. Compared with our previous DIBL-based analytical model [30] and the ballistic framework of [29], the present work makes three advances: (i) a unified SDT current model, based on a Wentzel–Kramers–Brillouin (WKB) approach of a parabolic potential barrier, that remains valid across all operating regions; (ii) an SDT-aware channel-charge and gate capacitance formulation; and (iii) a Verilog-A implementation verified at the circuit level in Simulation Program with Integrated Circuit Emphasis (SPICE). Then, an analytical compact model is validated against NEGF simulations. The remainder of this paper is organized as follows. Section 2 introduces the device structure and the drain current formulation. Section 3 derives the subband energy using a parabolic approximation and develops a unified tunneling transmission model. Section 4 presents the simulation results and discusses the model accuracy. Section 5 concludes the paper.

## 2. Modeling Theory

### 2.1. Device Structure

**Figure 1:**
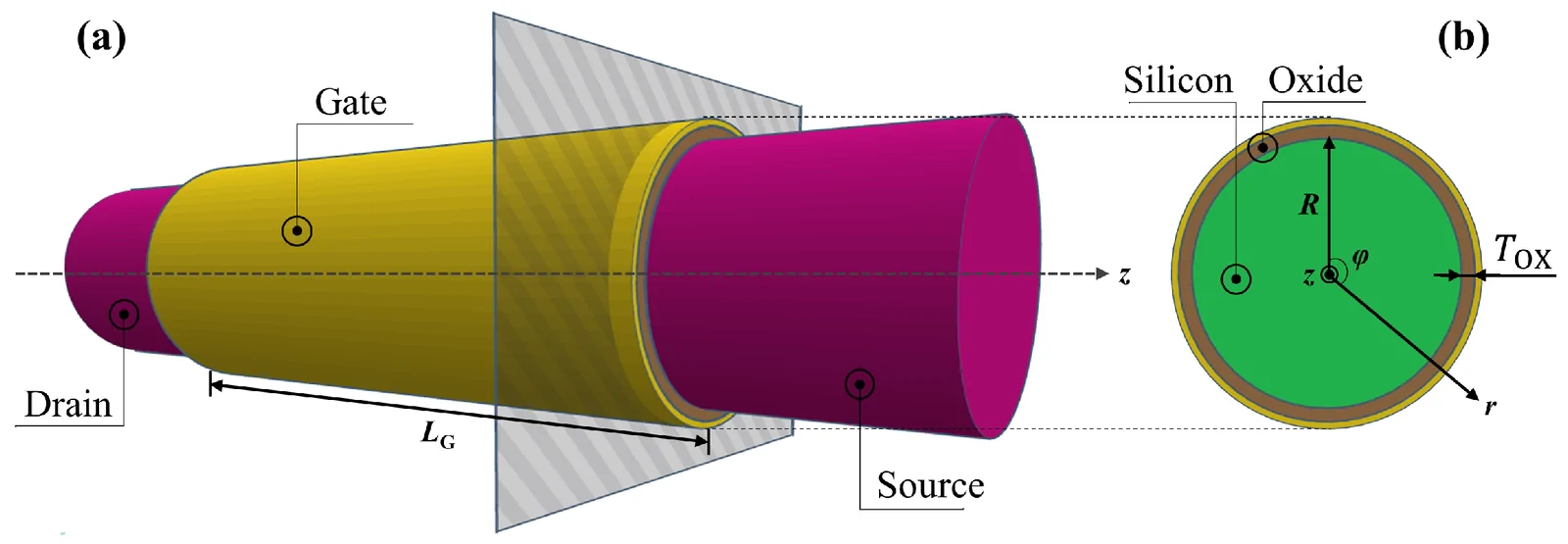
Schematic illustration of the p-type cylindrical nanowire GAA MOSFET structure along the (**a**) longitudinal (*z*-) and (**b**) transverse (*r*-) directions. The gray shading in (**a**) indicates the cross-sectional view in (**b**), which is taken in a plane perpendicular to the *z*-axis. The parameters *R*, $L_{G}$, and $T_{\mathrm{OX}}$ denote the nanowire radius, gate length, and oxide thickness, respectively, while *r*, φ, and *z* represent the radial, azimuthal, and transport coordinates.

Figure 1 illustrates the schematic structure of the p-type cylindrical nanowire GAA MOSFET investigated in this work. The device consists of a cylindrical silicon nanowire channel coaxially surrounded by a gate dielectric and a metal gate electrode. The main geometric parameters include the nanowire radius *R*, gate length $L_{G}$, and oxide thickness $T_{\mathrm{OX}}$. The source and drain regions are assumed to be heavily doped ($p^{+}$) silicon contacts, acting as ideal carrier reservoirs.

Assuming ideal carrier injection from the source and drain contacts, hole transport through the channel is described within the quasi-ballistic/ballistic transport regime. A cylindrical coordinate system is used, where *r*, φ, and *z* denote the radial, azimuthal, and transport directions, respectively. Due to the nanoscale dimensions of the device, quantum confinement in the radial direction and SDT along the transport direction are explicitly included in the proposed model.

### 2.2. Energy Level Profile

**Figure 2:**
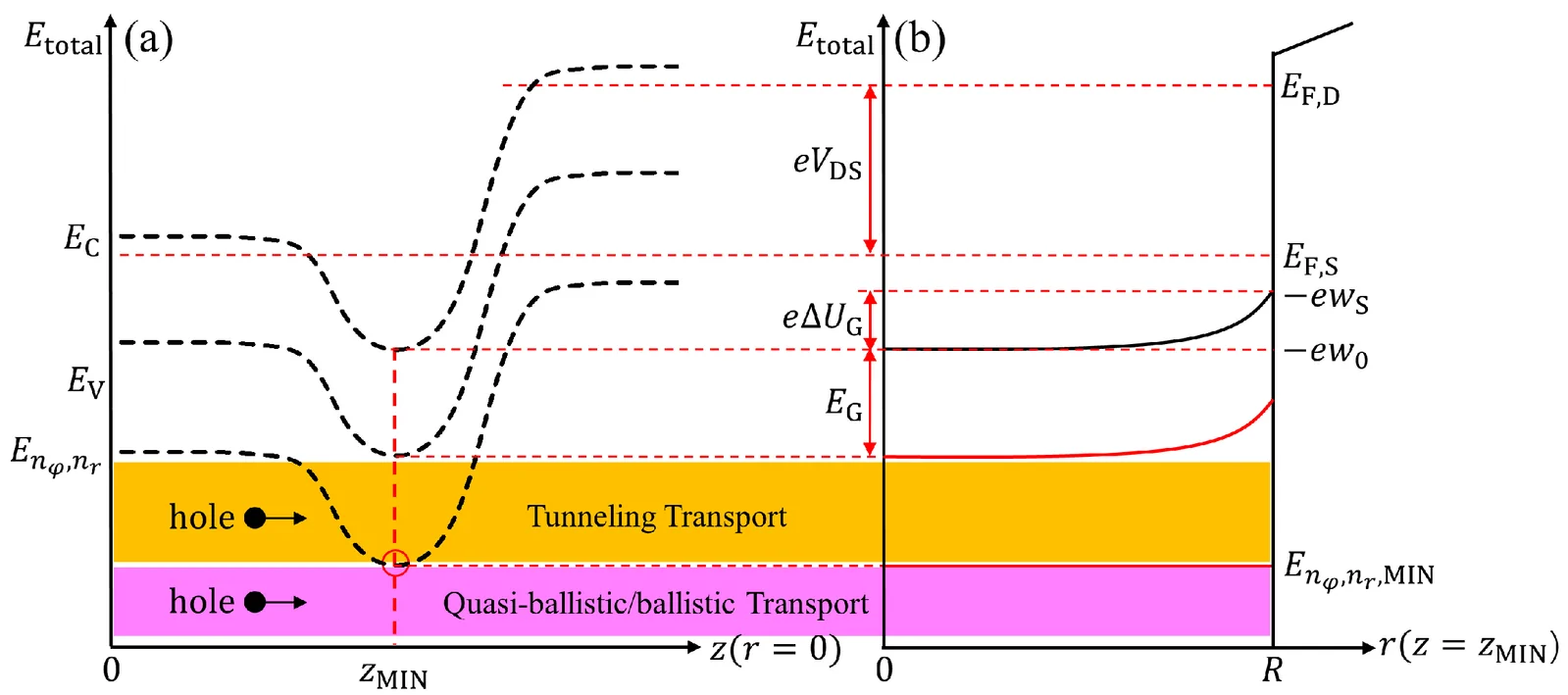
Schematic potential profiles and corresponding carrier transport mechanisms in the p-type nanowire GAA MOSFET. (**a**) Conduction- and valence-band-edge energy profiles and hole subband energy levels along the longitudinal (*z*-) direction at r=0. Black dashed lines denote $E_{C}$, $E_{V}$, and the subband energy level; the red circle marks the subband energy level $E_{n_{\varphi },n_{r},\mathrm{MIN}}$ at $z=z_{\mathrm{MIN}}$, and black solid circles denote holes and their transport direction. Holes with energies below the potential barrier undergo quasi-ballistic/ballistic transport, whereas those above the barrier contribute to SDT. (**b**) Conduction- and valence-band-edge energy profiles and the hole subband energy level along the transverse (*r*-) direction at $z=z_{\mathrm{MIN}}$; the black solid line denotes $E_{C}$, separated from $E_{V}$ by the bandgap $E_{G}$, and the red solid lines denote $E_{V}$ and the subband energy level.

Figure 2a,b illustrate the conduction- and valence-band edge energy profiles, together with the hole subband energy levels, along the longitudinal (*z*-) and transverse (*r*-) directions at r=0 and $z=z_{\mathrm{MIN}}$, respectively. Here, $E_{C}$ and $E_{V}$ denote the conduction-band and valence-band edges, respectively. The short-channel effect is assumed to be uniform in the transverse direction. As shown in Figure 2a, the minimum of the hole subband energy profile along the channel direction is located at $z=z_{\mathrm{MIN}}$ and is denoted as $E_{n_{\varphi },n_{r},\mathrm{MIN}}$, while $E_{\mathrm{total}}$ represents the total hole energy. The source and drain Fermi levels are denoted by $E_{F,S}$ and $E_{F,D}$, respectively, and the drain source voltage is defined as $V_{\mathrm{DS}}=\left(E_{F,S}-E_{F,D}\right)/e$. As shown in Figure 2b, the conduction-band edges at the channel center (r=0) and nanowire surface (r=R) are expressed as $-ew_{0}$ and $-ew_{S}$, respectively, where $w_{0}$ and $w_{S}$ are the corresponding electrostatic potentials. Then, the potential difference between the channel center and the surface, $w_{0}-w_{S}$, is described by the parameter $\Delta U_{G}$. Therefore, the hole subband energy level $E_{n_{\varphi },n_{r}}$, referenced to the source Fermi level $E_{F,S}$, is determined by the radial quantum confinement energy and the surface potential.

Based on our previous study [29,30], the hole subband energy $E_{n_{\varphi },n_{r}}\left(z\right)$ can be expressed as a function of $\Delta U_{G}\left(z\right)$. Accordingly, the subband energy level profile along the transport direction is given by

(1) $$E_{n_{\varphi },n_{r}}\left(z\right)=-e\cdot V_{\mathrm{GS}}^{*}-E_{G}-\frac{\hbar^{2}}{2m_{xh}^{r}}{\left(\frac{R_{n_{\varphi }}^{n_{r}}}{R}\right)}^{2}-\kappa \cdot \Delta U_{G}\left(z\right),$$

(2) $$V_{\mathrm{GS}}^{*}=V_{\mathrm{GS}}-\phi_{\mathrm{GC}}+w_{\mathrm{FB}},$$

(3) $$\kappa =e\left(H_{n_{\varphi },n_{r}}+\frac{1}{\beta^{2}}-1\right),$$

where $E_{G}$ denotes the bandgap between $E_{C}$ and $E_{V}$, $V_{\mathrm{GS}}$ represents the applied gate source voltage, $\phi_{\mathrm{GC}}$ is the work function difference between the gate and channel materials, and $w_{\mathrm{FB}}$ denotes the electrostatic potential corresponding to the conduction-band edge energy under the flat-band condition. The parameter $H_{n_{\varphi },n_{r}}$ represents the perturbation matrix element, and $m_{xh}^{r}$ denotes the radial effective mass of the confined holes. The subscript *x* identifies the hole band, including the heavy-hole (h), light-hole (l), and split-off (s) bands. In addition, $R_{n_{\varphi }}^{n_{r}}$ represents the $n_{r}$th zero of the first-kind Bessel function of order $n_{\varphi }$, as defined in [29]. The geometric scaling coefficient β is therefore expressed as

(4) $$\beta ={\left(\alpha_{\beta }+\frac{4\pi \epsilon_{\mathrm{CH}}}{C_{\mathrm{OX}}}\right)}^{-1/2},$$

where $\epsilon_{\mathrm{CH}}$ denotes the dielectric constant of the channel material, and $C_{\mathrm{OX}}$ represents the gate oxide capacitance per unit length, expressed as $2\pi \epsilon_{\mathrm{OX}}/\ln\left(1+T_{\mathrm{OX}}/R\right)$, with $\epsilon_{\mathrm{OX}}$ denoting the dielectric constant of the oxide material. The parameter $\alpha_{\beta }$ is an empirical fitting parameter without direct physical significance, and its value is listed in Table 1.

Substituting z=0 and $z=z_{\mathrm{MIN}}$ into Equation (1), the hole subband energy levels at the source boundary and the barrier minimum are obtained as

(5) $$E_{n_{\varphi },n_{r}}\left(0\right)=-e\cdot V_{\mathrm{GS}}^{*}-E_{G}-\frac{\hbar^{2}}{2m_{xh}^{r}}{\left(\frac{R_{n_{\varphi }}^{n_{r}}}{R}\right)}^{2}-\kappa \cdot \Delta U_{G}\left(0\right),$$

(6) $$E_{n_{\varphi },n_{r}}\left(z_{\mathrm{MIN}}\right)=-e\cdot V_{GS}^{*}-E_{G}-\frac{\hbar^{2}}{2m_{xh}^{r}}{\left(\frac{R_{n_{\varphi }}^{n_{r}}}{R}\right)}^{2}-\kappa \cdot \Delta U_{G}\left(z_{\mathrm{MIN}}\right).$$

Following the methodology established in our previous work [29,30], the expression for $\Delta U_{G}$ evaluated at $z=z_{\mathrm{MIN}}$, valid for all operating regions, is derived as

(7) $$\Delta U_{G}\left(z_{\mathrm{MIN}}\right)=-2\sqrt{\frac{1}{\alpha_{u2}}\cdot \ln\left[1+\exp\left(\alpha_{u2}\cdot A\cdot B\right)\right]}+\Delta U_{G}^{\left(1\right)}\left(\alpha_{u1}\right),$$

where $\Delta U_{G}^{\left(1\right)}$ is defined in [30], $\alpha_{u1}$ and $\alpha_{u2}$ are empirical fitting parameters without direct physical significance, where $\alpha_{u1}$ is introduced in $\Delta U_{G}^{\left(1\right)}$ and $\alpha_{u2}$ is employed in the overall expression of $\Delta U_{G}\left(z_{\mathrm{MIN}}\right)$. Their values are listed in Table 1. Accordingly, $\Delta U_{G}$ at z=0 is given by

(8) $$\Delta U_{G}\left(0\right)=-A-B,$$

where the coefficients *A* and *B* are expressed as

(9) $$A=-\rho \cdot \left\{\left(V_{\mathrm{bi}}-V_{\mathrm{GS}}^{*}\right)\left[1-\exp\left(-\frac{2\beta L_{G}}{R}\right)\right]+V_{\mathrm{DS}}\right\},$$

(10) $$B=-\rho \cdot \left\{\left(V_{\mathrm{bi}}-V_{\mathrm{GS}}^{*}\right)\left[\exp\left(\frac{2\beta L_{G}}{R}\right)-1\right]-V_{\mathrm{DS}}\right\},$$

(11) $$\rho =\frac{\beta^{2}}{1-\beta^{2}+\beta^{4}}\cdot \frac{1}{\exp\left(\frac{2\beta L_{G}}{R}\right)-\exp\left(-\frac{2\beta L_{G}}{R}\right)},$$

where $V_{\mathrm{bi}}$ is the built-in potential at the source/channel junction, and its values for different nanowire radii are listed in Table 2.

Moreover, to smoothly connect the analytical expressions in all operating regimes, a unified expression for $z_{\mathrm{MIN}}$ is adopted as

(12) $$z_{\mathrm{MIN}}^{\mathrm{uni}}=\frac{R}{4\beta }\cdot \ln\left\{1+\frac{1}{\alpha_{z}}\cdot \ln\left[1+\exp\left(\alpha_{z}\cdot \frac{B}{A}\right)\right]\right\},$$

where $\alpha_{z}$ is an empirical fitting parameter without direct physical significance. Its value is listed in Table 1.

### 2.3. Landauer’s Formula

**Figure 3:**
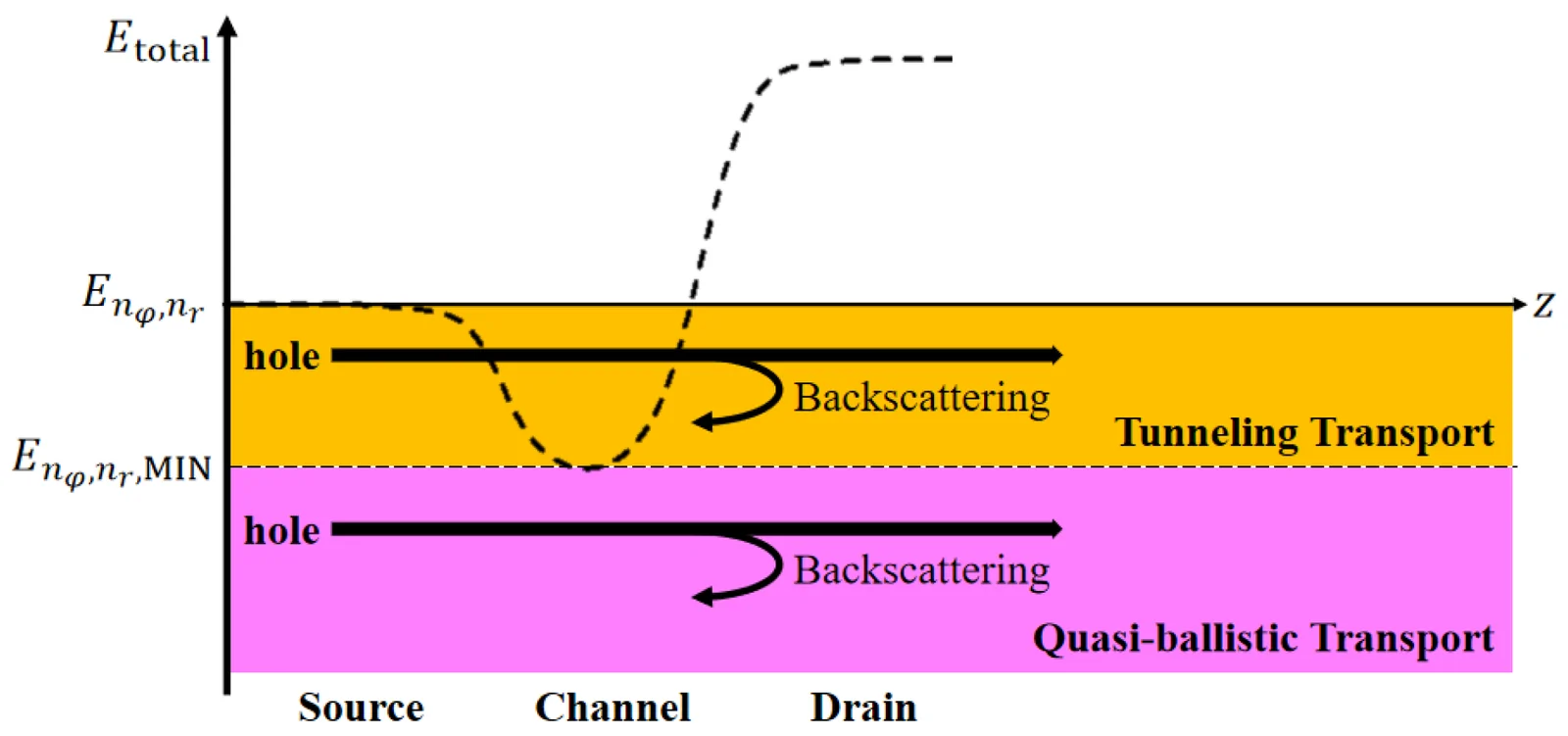
Schematic *E*-*z* profile and corresponding hole transport mechanisms between the source and drain. The dashed curve indicates the subband energy profile along the *z*-direction, and the horizontal dashed line marks the barrier-minimum energy level $E_{n_{\varphi },n_{r},\mathrm{MIN}}$, separating the tunneling-transport (SDT) and quasi-ballistic/ballistic-transport regimes. Holes with energies below the barrier minimum contribute to ballistic transport with channel backscattering, whereas holes with energies above the barrier minimum undergo SDT with backscattering. In the proposed model, backscattering is assumed to occur only after carriers pass through the barrier minimum.

In this work, quasi-ballistic transport is described using a ballistic transport model that includes the channel backscattering effect. As illustrated in Figure 3, the hole transport mechanisms between the source and drain are determined by the comparison between the total hole energy and the subband energy level profile along the channel direction. For holes with total energies above the barrier minimum, SDT occurs through the potential barrier. An effective transmission probability is introduced to account for both quantum tunneling and channel backscattering during transport. In contrast, holes with total energies below the barrier minimum overcome the potential barrier and contribute to quasi-ballistic transport with channel backscattering included [29,31,32,33]. In the proposed model, backscattering is assumed to occur only after carriers traverse the barrier minimum.

Quasi-ballistic transport is expected to dominate carrier transport in short-channel devices [30,33,34,35]. However, as the channel length is further scaled to the ultra-short-channel regime, SDT becomes increasingly important and cannot be neglected. Therefore, by considering both thermionic quasi-ballistic transport and SDT, the hole current in the p-type nanowire GAA MOSFET is formulated within the Landauer transport framework as

(13) $$\begin{array}{c} I_{\mathrm{SD}}=\frac{e}{\pi \hbar }\sum_{n_{\varphi },n_{r}}\sum_{n_{\nu }}\int_{-\infty }^{0}dE_{z}\cdot g_{n_{\nu }}\cdot T_{n_{\varphi },n_{r}}^{\mathrm{eff}}\left(E_{z}\right)\cdot \left[f_{h}\left(E_{\mathrm{total}},E_{F,S}\right)-f_{h}\left(E_{\mathrm{total}},E_{F,D}\right)\right], \end{array}$$

(14) $$E_{\mathrm{total}}=E_{n_{\varphi },n_{r}}\left(0\right)+E_{z},$$

(15) $$f_{h}\left(E_{\mathrm{total}},E_{F,x}\right)={\left[1+\exp\left(\frac{E_{F,x}-E_{\mathrm{total}}}{k_{B}T}\right)\right]}^{-1},$$

(16) $$E_{F,D}=E_{F,S}-eV_{\mathrm{DS}},$$

where *e*, *ħ*, and $k_{B}$ denote the elementary charge, reduced Planck constant, and Boltzmann constant, respectively; $g_{n_{\nu }}$ is the valley degeneracy for the valley index $n_{\nu }$; $E_{\mathrm{total}}$ is the total hole energy; and $E_{z}$ is the kinetic energy along the transport direction. Furthermore, $E_{n_{\varphi },n_{r}}$ denotes the hole subband energy level with angular and radial quantum numbers $n_{\varphi }$ and $n_{r}$, respectively, while $f_{h}$ is the Fermi–Dirac distribution function for holes. The effective transmission coefficient $T_{n_{\varphi },n_{r}}^{\mathrm{eff}}\left(E_{z}\right)$ represents the transmission probability of holes with kinetic energy $E_{z}$ in the $\left(n_{\varphi },n_{r}\right)$ subband, including the effects of SDT, thermionic ballistic transport, and channel backscattering. Then, it is given by [36]

(17) $$T_{n_{\varphi },n_{r}}^{\mathrm{eff}}\left(E_{z}\right)=\frac{T_{n_{\varphi },n_{r}}\left(E_{z}\right)\cdot \left(1-R_{\mathrm{ref}}\right)}{1-R_{\mathrm{ref}}+T_{n_{\varphi },n_{r}}\left(E_{z}\right)\cdot R_{\mathrm{ref}}},$$

where $T_{n_{\varphi },n_{r}}\left(E_{z}\right)$ denotes the transmission coefficient of holes in the $\left(n_{\varphi },n_{r}\right)$ subband between the source and drain, including both SDT and thermionic quasi-ballistic transport, and $R_{\mathrm{ref}}$ is the channel backscattering coefficient assumed to be constant. Using the WKB approximation for the tunneling regime above the barrier minimum and unity transmission for the ballistic regime below the barrier minimum, $T_{n_{\varphi },n_{r}}\left(E_{z}\right)$ is expressed as

(18) $$T_{n_{\varphi },n_{r}}\left(E_{z}\right)=\left\{\begin{array}{cc} 1, & E_{z}<E_{n_{\varphi },n_{r}}\left(z_{\mathrm{MIN}}\right),\\ \exp\left[-\frac{2}{\hbar }\int_{z_{1}}^{z_{2}}\sqrt{2m_{xh}^{z}\left(E_{\mathrm{total}}-E_{n_{\varphi },n_{r}}\left(z\right)\right)}dz\right], & E_{z}>E_{n_{\varphi },n_{r}}\left(z_{\mathrm{MIN}}\right), \end{array}\right.$$

where $z_{1}$ and $z_{2}$ denote the classical turning points of the tunneling barrier for a given kinetic energy $E_{z}$, obtained from the energy conservation relation in Equation (14) between the total hole energy $E_{\mathrm{total}}$ and the subband energy profile at the source $E_{n_{\varphi },n_{r}}\left(0\right)$. Furthermore, $m_{xh}^{z}$ denotes the hole effective mass along the transport direction.

## 3. Modeling Method

In this study, hole transport is divided into two components according to the carrier energy relative to the barrier minimum. Holes with energies above the barrier minimum contribute to SDT, whereas those with energies below the barrier minimum overcome the potential barrier through quasi-ballistic transport. To enable a direct comparison with the purely ballistic NEGF benchmark, the channel backscattering coefficient is set to $R_{\mathrm{ref}}=0$ throughout this work, corresponding to the ideal ballistic limit. A nonzero $R_{\mathrm{ref}}$ modifies the effective transmission probability through Equation (17) and allows the same mathematical framework to describe quasi-ballistic transport. However, no quasi-ballistic reference data are available from the present NEGF setup, and all validation reported in Section 4 is therefore limited to the ballistic regime.

Furthermore, only the lowest subband ($n_{\varphi }=0$, $n_{r}=1$) is considered throughout this work, assuming that strong quantum confinement suppresses the occupation of higher-energy subbands within the investigated operating region. Accordingly, only the heavy-hole band with a valley degeneracy of one is included in the model. In addition, quantum reflection at the channel potential barrier is neglected when evaluating the transmission probability. Under these assumptions, the proposed compact model provides a unified description of hole transport through and over the source-to-drain potential barrier.

### 3.1. Parabolic Approximation for Energy Level

According to our previous study [29], the potential profile of ultra-short-channel nanowire MOSFETs can be well approximated by a quadratic function along the transport direction. Therefore, two characteristic positions, z=0 and $z=z_{\mathrm{MIN}}$, are considered in this work, representing the source reference point and the barrier minimum, respectively. Under the ultra-short-channel approximation, the lowest hole subband energy level profile, $E_{0,1}\left(z\right)$, along the transport direction is described by a parabolic function, denoted as $E_{0,1,B}\left(z\right)$, which is given by

(19) $$\begin{array}{cc} E_{0,1,B}\left(z\right) & =a_{B}\cdot {\left(1-\frac{z}{z_{\mathrm{MIN}}^{\mathrm{uni}}}\right)}^{2}+b_{B}, \end{array}$$

(20) $$\begin{array}{cc} a_{B} & =E_{0,1}\left(0\right)-E_{0,1}\left(z_{\mathrm{MIN}}^{\mathrm{uni}}\right), \end{array}$$

(21) $$\begin{array}{cc} b_{B} & =E_{0,1}\left(z_{\mathrm{MIN}}^{\mathrm{uni}}\right). \end{array}$$

### 3.2. Transmission Coefficient

**Figure 4:**
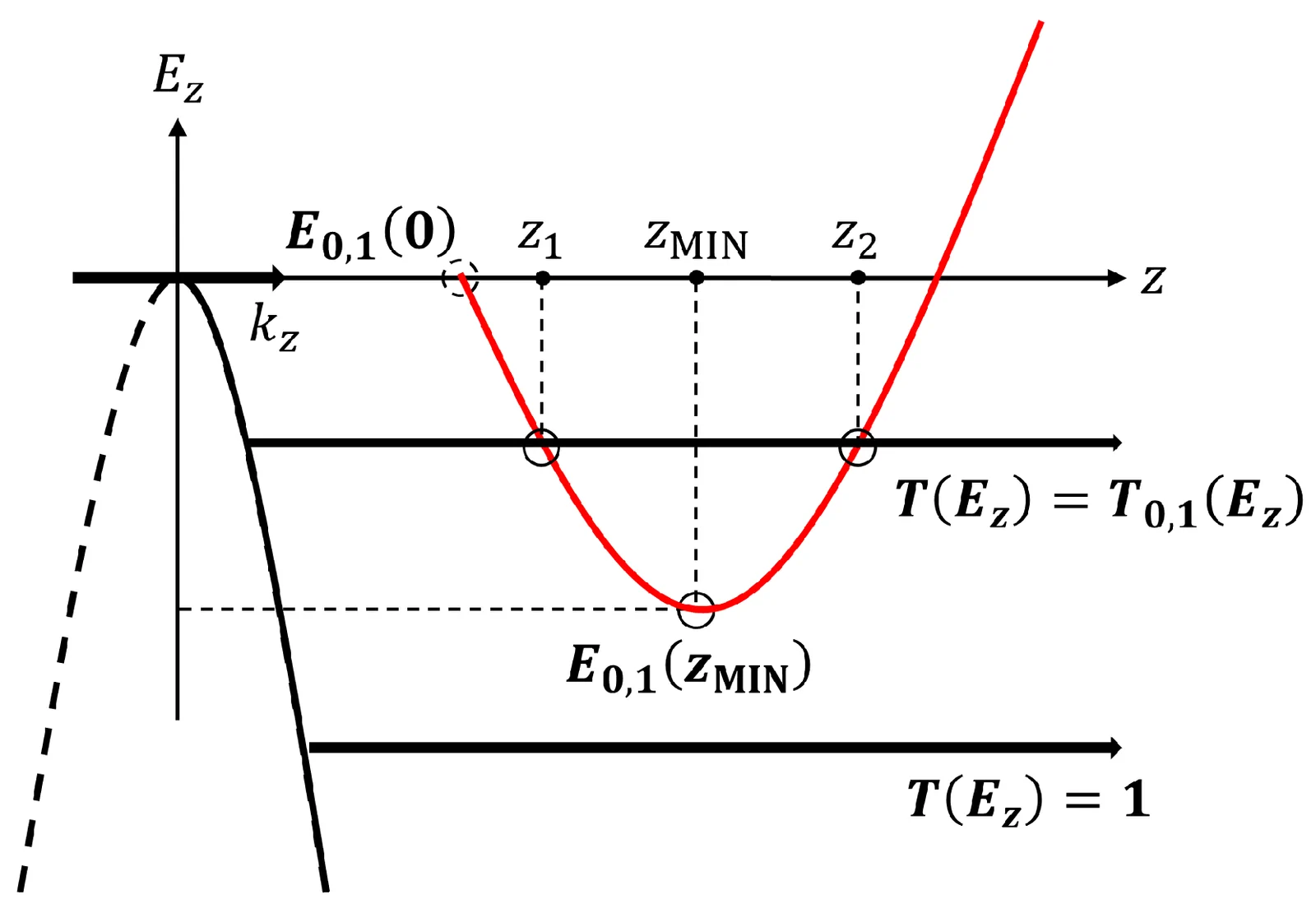
Schematic diagram of hole transport between the source and drain as a function of the total hole energy. The red curve indicates the subband energy profile (parabolic barrier) along the *z*-direction, and the black dashed lines mark the classical turning points $z_{1}$ and $z_{2}$, as well as the barrier-minimum energy level $E_{0,1}\left(z_{\mathrm{MIN}}\right)$ at $z=z_{\mathrm{MIN}}$, with circles indicating the corresponding energy levels at these *z*-positions. The black parabolic curve indicates the parabolic dispersion relation between the hole wave vector $k_{z}$ and the kinetic energy $E_{z}$. Both thermionic quasi-ballistic transport over the channel potential barrier and source-to-drain quantum tunneling through the barrier are considered: the black solid arrow labeled $T(E_{z})=1$ denotes the ballistic transmission, and the black solid arrow labeled $T\left(E_{z}\right)=T_{0,1}\left(E_{z}\right)$ denotes the direction and magnitude of the tunneling transmission.

Figure 4 schematically illustrates the resulting hole transport picture as a function of the total hole energy, showing the classical turning points $z_{1}$ and $z_{2}$, the barrier-minimum energy level $E_{0,1}\left(z_{\mathrm{MIN}}\right)$, and the parabolic dispersion relation between $k_{z}$ and $E_{z}$. Based on Equation (18), the transmission coefficient in the SDT regime is evaluated for the energy range satisfying $E_{0,1}\left(0\right)>E_{z}>E_{0,1}\left(z_{\mathrm{MIN}}\right)$. By substituting the parabolic approximation of the lowest-subband energy level profile in Equation (19) into Equation (18), the tunneling transmission coefficient $T_{0,1}\left(E_{z}\right)$ is obtained as

(22) $$T_{0,1}\left(E_{z}\right)=\exp\left[-\frac{\pi }{\hbar }\cdot \sqrt{\frac{2m_{\mathrm{hh}}^{z}}{a_{B}}}\left(E_{z}+a_{B}\right)\cdot z_{\mathrm{MIN}}^{\mathrm{uni}}\right].$$

Then, Equation (22) can be rewritten in the following unified form, which is valid over the entire operating regions and energy range:

(23) $$\begin{array}{c} T_{0,1}^{\mathrm{uni}}\left(E_{z}\right)=\exp\left\{-\frac{1}{\alpha_{T}}\cdot \ln\left[1+\exp\left(\alpha_{T}\cdot \frac{\pi }{\hbar }\sqrt{\frac{2m_{\mathrm{hh}}^{z}}{a_{B}}}\left(E_{z}+a_{B}\right)\cdot z_{\mathrm{MIN}}^{\mathrm{uni}}\right)\right]\right\}, \end{array}$$

where $\alpha_{T}$ is an empirical fitting parameter, and its fitted value is listed in Table 1. Since $E_{0,1}\left(0\right)$ and $T_{0,1}^{\mathrm{uni}}$ are defined over all transport regimes using Equations (5) and (23), respectively, the total drain current is expressed as

(24) $$\begin{array}{cc} I_{\mathrm{SD}}= & \frac{e}{\pi \hbar }\int_{-\infty }^{0}dE_{z}\cdot T_{0,1}^{\mathrm{uni}}\left(E_{z}\right)\\ \; & \;\times \left\{\frac{1}{1+\exp\left[\frac{-E_{z}-E_{0,1}\left(0\right)}{k_{B}T}\right]}-\frac{1}{1+\exp\left[\frac{-E_{z}-E_{0,1}\left(0\right)-eV_{\mathrm{DS}}}{k_{B}T}\right]}\right\}. \end{array}$$

### 3.3. Channel Hole Charge Line Density

In the ultra-short-channel regime, SDT makes a significant contribution to the channel hole density and cannot be neglected, particularly in the subthreshold region. Accordingly, the total channel hole density is assumed to consist of two components: the thermionic hole density from ballistic transport below the barrier minimum and the tunneling hole density from source-to-drain quantum tunneling through the potential barrier [37,38]. The one-dimensional hole charge line density is calculated by summing the contributions from the forward- and backward-propagating states, which are occupied according to $E_{F,S}$ and $E_{F,D}$, respectively. Based on quantum statistics, the hole charge line density is obtained by integrating the density of states weighted by the occupation probability and the energy-dependent transmission coefficient. The integration is performed over the kinetic energy $E_{z}$, which is referenced to the lowest subband energy level at the source, $E_{0,1}\left(0\right)$. Therefore, the total one-dimensional hole charge line density is given by

(25) $$\begin{array}{cc} Q_{\mathrm{qh}}= & \frac{e}{\pi \hbar }\sqrt{\frac{m_{\mathrm{hh}}^{z}}{2}}\cdot \int_{-\infty }^{0}dE_{z}\cdot T_{0,1}^{\mathrm{uni}}\left(E_{z}\right)\cdot {\left(-E_{z}\right)}^{-1/2}\\ \; & \;\times \left\{\frac{1}{1+\exp\left[\frac{-E_{z}-E_{0,1}\left(0\right)}{k_{B}T}\right]}+\frac{1}{1+\exp\left[\frac{-E_{z}-E_{0,1}\left(0\right)-eV_{\mathrm{DS}}}{k_{B}T}\right]}\right\}. \end{array}$$

Equations (24) and (25) are written in their general two-reservoir form and retain both Fermi–Dirac occupation terms, so that the drain current vanishes correctly at equilibrium ($V_{\mathrm{DS}}=0$) and so that the analytical expressions of Section 3.5 can be derived from them. Under the operating condition $|eV_{\mathrm{DS}}|\;\gg k_{B}T$ used throughout this work, however, the drain-injected hole contribution is exponentially suppressed, and the integrands of Equations (24) and (25) are evaluated numerically with the source-injected term only [39]. The same approximation is applied to the analytical tunneling current derived in Section 3.5, whereas the analytical thermionic current of Equation (27) retains the drain-side term in full. Consequently, the drain-side term of Equations (24) and (25) does not affect any quantity reported in Section 4.

It should be noted that, with SDT considered, the thermionic component can no longer be described by the conventional Fermi–Dirac integral used in ballistic models without tunneling [40]. In the conventional formulation the channel charge is evaluated at the top of the barrier (T=1), so that the one-dimensional density of states is referenced to $E_{0,1}\left(z_{\mathrm{MIN}}\right)$ and is proportional to $|E_{\mathrm{total}}-E_{0,1}\left(z_{\mathrm{MIN}}\right){|}^{-1/2}$. In the present model, the flux-injected charge is instead evaluated at the source reference point, so that the density of states is referenced to $E_{0,1}\left(0\right)$ and is proportional to $|E_{\mathrm{total}}-E_{0,1}{\left(0\right)|}^{-1/2}$, which reduces to $|E_{z}{|}^{-1/2}$ through the energy relation of Equation (14) and appears in this form in Equation (25). In addition, the occupation of each state is additionally weighted by the energy-dependent transmission coefficient $T_{0,1}^{\mathrm{uni}}\left(E_{z}\right)$. As a result, Equation (25) cannot be reduced to the conventional Fermi integral: the thermionic and tunneling contributions are not evaluated separately, but are combined in a single integral in which $T_{0,1}^{\mathrm{uni}}\left(E_{z}\right)$ approaches unity below the barrier minimum and decreases within the tunneling window above it.

### 3.4. Capacitance Characteristics

Using the one-dimensional hole charge line density derived in Equation (25), the gate capacitance per unit length, $C_{q}$, is obtained by differentiating the total channel charge with respect to the gate voltage $V_{\mathrm{GS}}$ and is expressed as

(26) $$C_{q}=\frac{\partial Q_{\mathrm{qh}}}{\partial V_{\mathrm{GS}}}.$$

Since the differentiation in Equation (26) is performed with respect to the externally applied gate voltage, the effect of the gate oxide voltage drop is naturally included in the charge response. Therefore, this definition represents the effective gate capacitance of the device, as discussed further in Section 4. Moreover, when SDT is considered, the conventional quantum capacitance definition based on the one-dimensional density of states and the source Fermi–Dirac distribution [39,41,42] is no longer accurate for ultra-short-channel devices, as the energy-dependent transmission coefficient affects the carrier distribution. In contrast, the derivative-based definition remains valid for both thermionic ballistic and SDT transport regimes.

### 3.5. Analytical Source-to-Drain Current Expressions

Following our previous formulation [29], the drain current due to thermionic ballistic transport is written as

(27) $$I_{\mathrm{SD}}^{\mathrm{therm}}=\frac{ek_{B}T}{\pi \hbar }\ln\left[\frac{1+\exp\left(\frac{E_{0,1}\left(z_{\mathrm{MIN}}\right)}{k_{B}T}\right)}{1+\exp\left(\frac{E_{0,1}\left(z_{\mathrm{MIN}}\right)+eV_{\mathrm{DS}}}{k_{B}T}\right)}\right].$$

For a sufficiently large drain bias satisfying $|eV_{\mathrm{DS}}|\;\gg k_{B}T$, the contribution of drain-injected holes can be neglected. Furthermore, assuming a Boltzmann distribution for holes and substituting Equation (22) into Equation (13), the drain current due to SDT is simplified as

(28) $$I_{\mathrm{SD}}^{\mathrm{tun}}=\frac{ek_{B}T}{\pi \hbar }\cdot \frac{\exp\left(G\right)-\exp\left(G-F\cdot H\right)}{F}\cdot f_{\mathrm{fit}},$$

(29) $$F=\left(-\frac{\pi }{\hbar }\sqrt{\frac{2m_{\mathrm{hh}}^{z}}{a_{B}}}\cdot z_{\mathrm{MIN}}^{\mathrm{uni}}\right)\cdot k_{B}T+1,$$

(30) $$G=\left(-\frac{\pi }{\hbar }\sqrt{\frac{2m_{\mathrm{hh}}^{z}}{a_{B}}}\cdot z_{\mathrm{MIN}}^{\mathrm{uni}}\right)\cdot a_{B}+\frac{E_{0,1}\left(0\right)}{k_{B}T},$$

(31) $$H=\frac{a_{B}}{k_{B}T},$$

(32) $$f_{\mathrm{fit}}={\left\{1+\exp\left[-\alpha_{\mathrm{fit}}\cdot \left(V_{\mathrm{GS}}-\beta_{\mathrm{fit}}\right)\right]\right\}}^{-1}\cdot \left[1-\exp\left(\frac{eV_{\mathrm{DS}}}{k_{B}T}\right)\right],$$

where $f_{\mathrm{fit}}$ denotes the smoothing function introduced to correct the overestimated tunneling current calculated from the WKB-based transmission coefficient in Equation (22), which exceeds unity in the inversion region. The parameters $\alpha_{\mathrm{fit}}$ and $\beta_{\mathrm{fit}}$ are empirical fitting parameters without direct physical meaning, and their values are summarized in Table 1. In addition, although the drain-injected hole density is neglected under a large drain-bias condition, the factor $1-\exp(eV_{\mathrm{DS}}/k_{B}T)$ is retained in Equation (32) as a correction term to ensure zero current at equilibrium ($V_{\mathrm{DS}}=0$). Then, combining the thermionic and tunneling components, the total drain current is written as

(33) $$I_{\mathrm{SD}}^{\mathrm{total}}=I_{\mathrm{SD}}^{\mathrm{tun}}+I_{\mathrm{SD}}^{\mathrm{therm}}.$$

## 4. Results and Discussion

In this section, the proposed compact model is benchmarked against NEGF simulations based on a multi-subband transport framework [43]. The NEGF calculations are performed using a self-consistent Poisson–NEGF approach within the effective-mass approximation. The nanowire transport direction is along the 〈100〉 orientation, and the effective mass is calibrated to reproduce the corresponding NEGF subband structure. For simplicity, a single-valley heavy-hole (HH) model with an isotropic effective mass is adopted, where the radial and transport-direction masses are assumed to be identical and equal to the value listed in Table 2. Therefore, the valence-band anisotropy is not considered in the proposed model. Since the lowest HH subband dominates hole transport in the investigated devices, only the lowest subband ($n_{\varphi }=0$, $n_{r}=1$) with a single-valley approximation ($g_{n_{\nu }}=1$) is included. In contrast, the NEGF simulations include multiple subbands within the same effective-mass framework. Although the subband descriptions differ, both approaches use the same calibrated effective mass and HH band model, ensuring a consistent comparison under their respective assumptions.

The investigated devices have nanowire radii of R=1.5, 2.0, and 2.5 nm and channel lengths of $L_{G}=4$ and 6 nm. The source/drain regions contain 10-nm-long p^+^-doped extensions with a uniform doping concentration of $1\times 10^{20}$ cm^−3^, and the oxide thickness is fixed at $T_{\mathrm{OX}}=0.5$ nm. The channel is assumed to be intrinsic. Unless otherwise specified, the remaining parameters are listed in Table 2. The gate work function is set to 4.8 eV, and the drain source voltage follows the IRDS roadmap [1]. The bandgap and built-in potential values used in the compact model are extracted from the NEGF simulations and listed in Table 2. The source Fermi level $E_{F,S}$ is used as the energy reference and set to zero in the NEGF calculations. Since the NEGF simulations assume fully ballistic hole transport, $R_{\mathrm{ref}}=0$ is used throughout this work to enable a direct comparison between the compact model and NEGF results. Quantum reflection at the channel potential barrier and parasitic source/drain resistances are neglected. The complete simulation input files are available in the GitHub repository provided in the Data Availability Statement. All NEGF simulations are performed using Silvaco ATLAS v5.22.1.R within the DeckBuild environment.

The NEGF simulations serve two purposes in the present study: they provide the physical parameters used in the compact model, such as the bandgap and built-in potential, and they provide the reference results for benchmarking the compact model. The global empirical fitting parameters are determined once and then kept unchanged across the investigated device geometries and bias conditions. The same set of NEGF simulations is used both for parameter extraction or calibration and for benchmarking the compact model, so the extraction and validation data are not independent. Therefore, the validation presented here evaluates the internal consistency of the model and the transferability of a single global parameter set across the investigated geometries and bias conditions, rather than its predictive capability on unseen devices. Validation against an independent dataset is left for future work.

The results presented in this section show good agreement between the proposed compact model and NEGF simulation results over the investigated range of channel lengths, nanowire radii, and bias conditions, demonstrating the validity of the HH-based analytical framework for ultra-short-channel p-type nanowire GAA MOSFETs.

**Figure 5:**
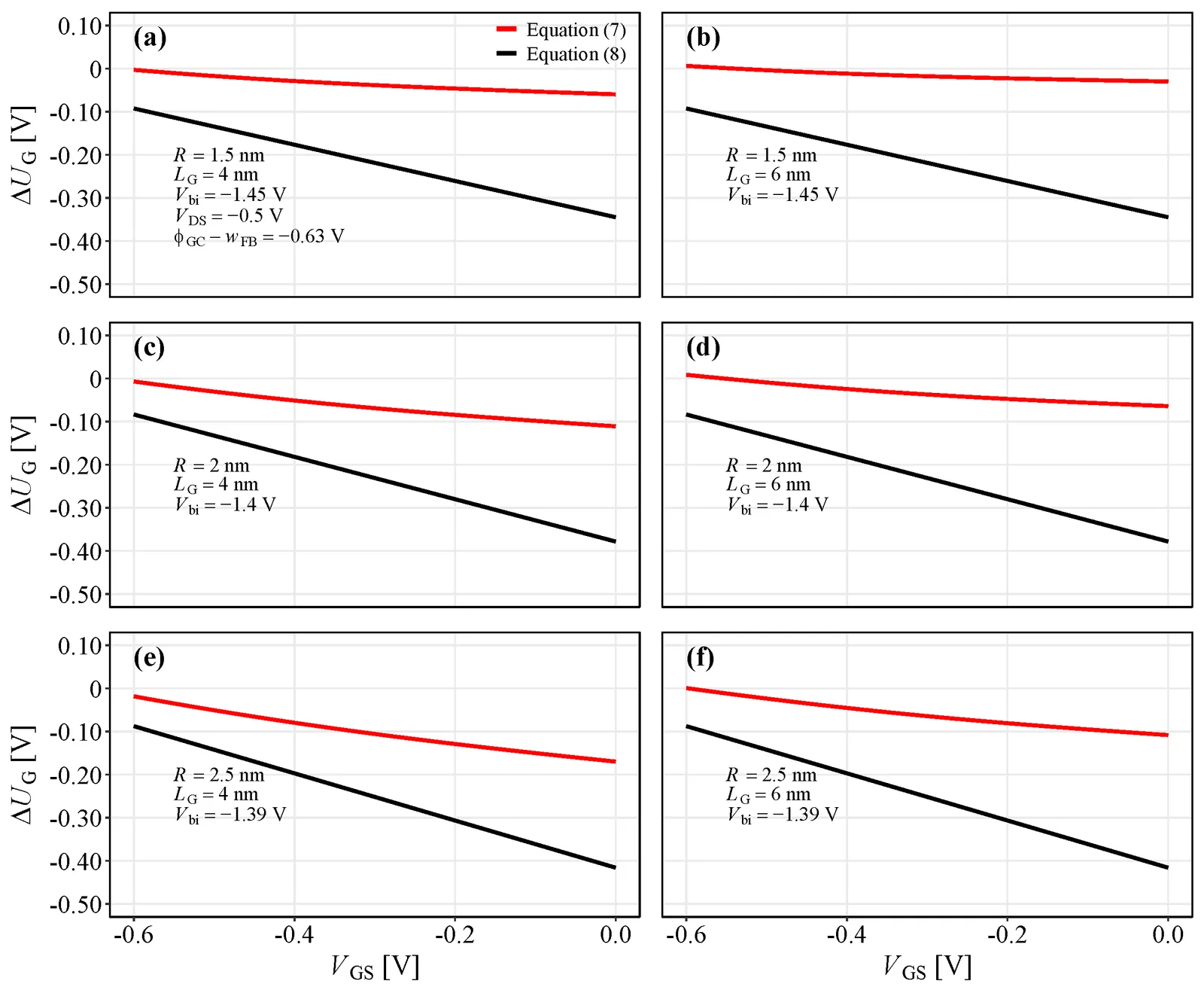
Comparison of the calculated $\Delta U_{G}\left(z_{\mathrm{MIN}}\right)$ and $\Delta U_{G}\left(0\right)$, obtained from Equations (7) and (8) and shown as solid red and solid black lines, respectively, as a function of the gate voltage. (**a**,**b**) R=1.5 nm; (**c**,**d**) R=2 nm; (**e**,**f**) R=2.5 nm. The left column corresponds to $L_{G}=4$ nm and the right column to $L_{G}=6$ nm. All panels are calculated at $V_{\mathrm{DS}}=-0.5$ V and $\phi_{\mathrm{GC}}-w_{\mathrm{FB}}=-0.63$ V; the built-in potential of each nanowire radius is annotated in the corresponding panel.

Figure 5 shows $\Delta U_{G}$ at the barrier minimum ($z=z_{\mathrm{MIN}}$, solid red line) and the source reference point (z=0, solid black line), calculated using Equations (7) and (8), respectively. In every panel, $\Delta U_{G}$ remains negative over the entire operating range. As the gate length $L_{G}$ decreases, the DIBL effect becomes stronger, leading to a more negative $\Delta U_{G}\left(z_{\mathrm{MIN}}\right)$. In contrast, reducing the nanowire radius R enhances quantum confinement, which suppresses the DIBL effect and makes $\Delta U_{G}\left(z_{\mathrm{MIN}}\right)$ less negative. The left and right columns correspond to $L_{G}=4$ nm and $L_{G}=6$ nm, respectively, while the three rows correspond to R=1.5,2.0, and 2.5 nm, respectively, allowing the effects of channel length and radial confinement to be directly compared.

**Figure 6:**
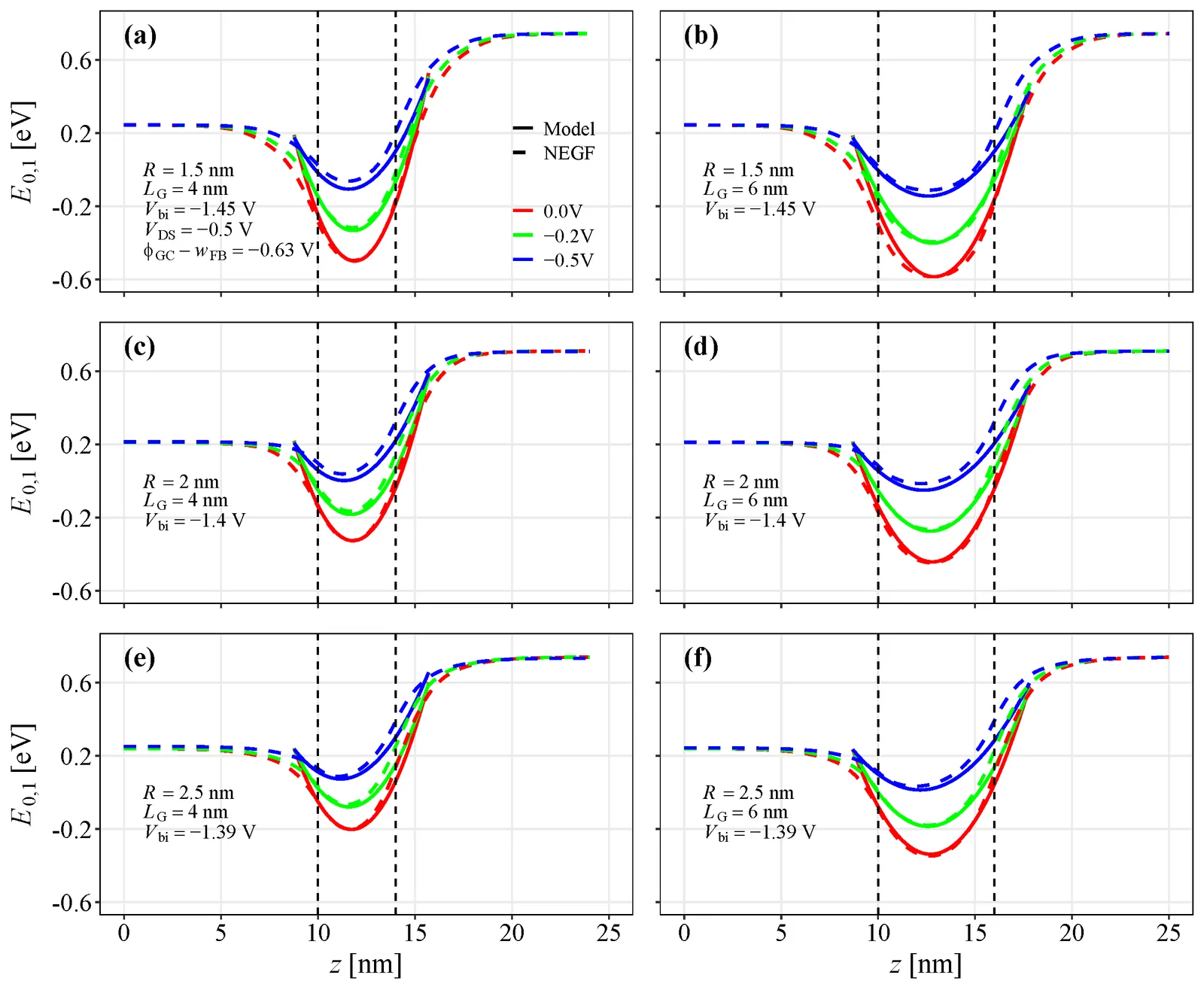
Comparison of the lowest hole subband energy profiles calculated by the compact model using Equation (19) (solid lines) and the NEGF simulations (dashed lines) under different gate bias conditions. The vertical dashed lines indicate the source/channel and channel/drain interfaces. (**a**,**b**) R=1.5 nm; (**c**,**d**) R=2 nm; (**e**,**f**) R=2.5 nm. The left column corresponds to $L_{G}=4$ nm and the right column to $L_{G}=6$ nm. All panels are calculated at $V_{\mathrm{DS}}=-0.5$ V and $\phi_{\mathrm{GC}}-w_{\mathrm{FB}}=-0.63$ V; the built-in potential of each nanowire radius is annotated in the corresponding panel.

Figure 6 compares the lowest hole subband energy level profiles along the channel obtained from NEGF simulations (dashed lines) with those calculated by the proposed compact model (solid lines) using Equation (19). The results are presented for gate biases of $V_{\mathrm{GS}}=0$, −0.2, and −0.5 V, from bottom to top in the figure. The effective source- and drain-side extensions are determined by comparing the compact-model subband energy profiles of Equation (19) with the corresponding NEGF profiles at the IRDS supply voltage of $V_{\mathrm{DS}}=-0.5$ V, and are chosen so that the analytical profile follows the NEGF profile over the channel region. The source-side extension is fixed at $\Delta L_{S}=1$ nm for all investigated device geometries. The drain-side extension is $\Delta L_{D}=1.8$ nm for $L_{G}=4$ nm and $\Delta L_{D}=2$ nm for $L_{G}=6$ nm. The two values differ by only 0.2 nm, which is small compared with the 1–2 nm scale of the extensions themselves, so that the drain-side extension is essentially geometry-independent within the accuracy of the present approach. These extensions are treated as model parameters rather than fitting parameters and are listed in Table 2. Once determined at the IRDS supply voltage, they are kept fixed for all subsequent gate bias conditions and are not re-adjusted. They effectively capture the source/drain depletion effects and barrier modulation through an extended analytical channel length, while avoiding explicit calculation of the detailed junction electrostatics.

**Figure 7:**
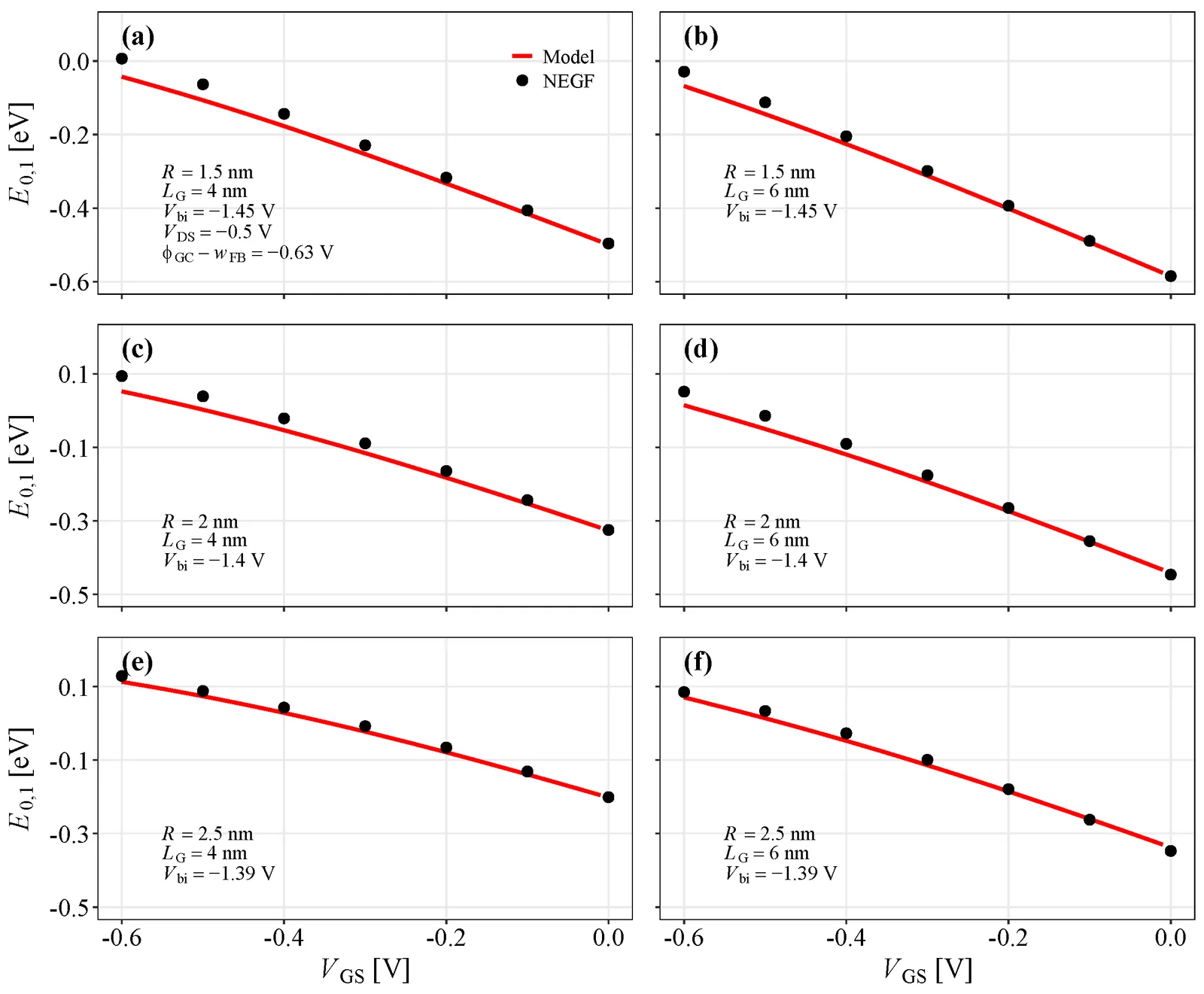
Comparison of the lowest hole subband energy at the barrier minimum as a function of gate voltage between the NEGF simulations (symbols) and the proposed compact model (solid lines). (**a**,**b**) R=1.5 nm; (**c**,**d**) R=2 nm; (**e**,**f**) R=2.5 nm. The left column corresponds to $L_{G}=4$ nm and the right column to $L_{G}=6$ nm. All panels are calculated at $V_{\mathrm{DS}}=-0.5$ V and $\phi_{\mathrm{GC}}-w_{\mathrm{FB}}=-0.63$ V; the built-in potential of each nanowire radius is annotated in the corresponding panel.

**Figure 8:**
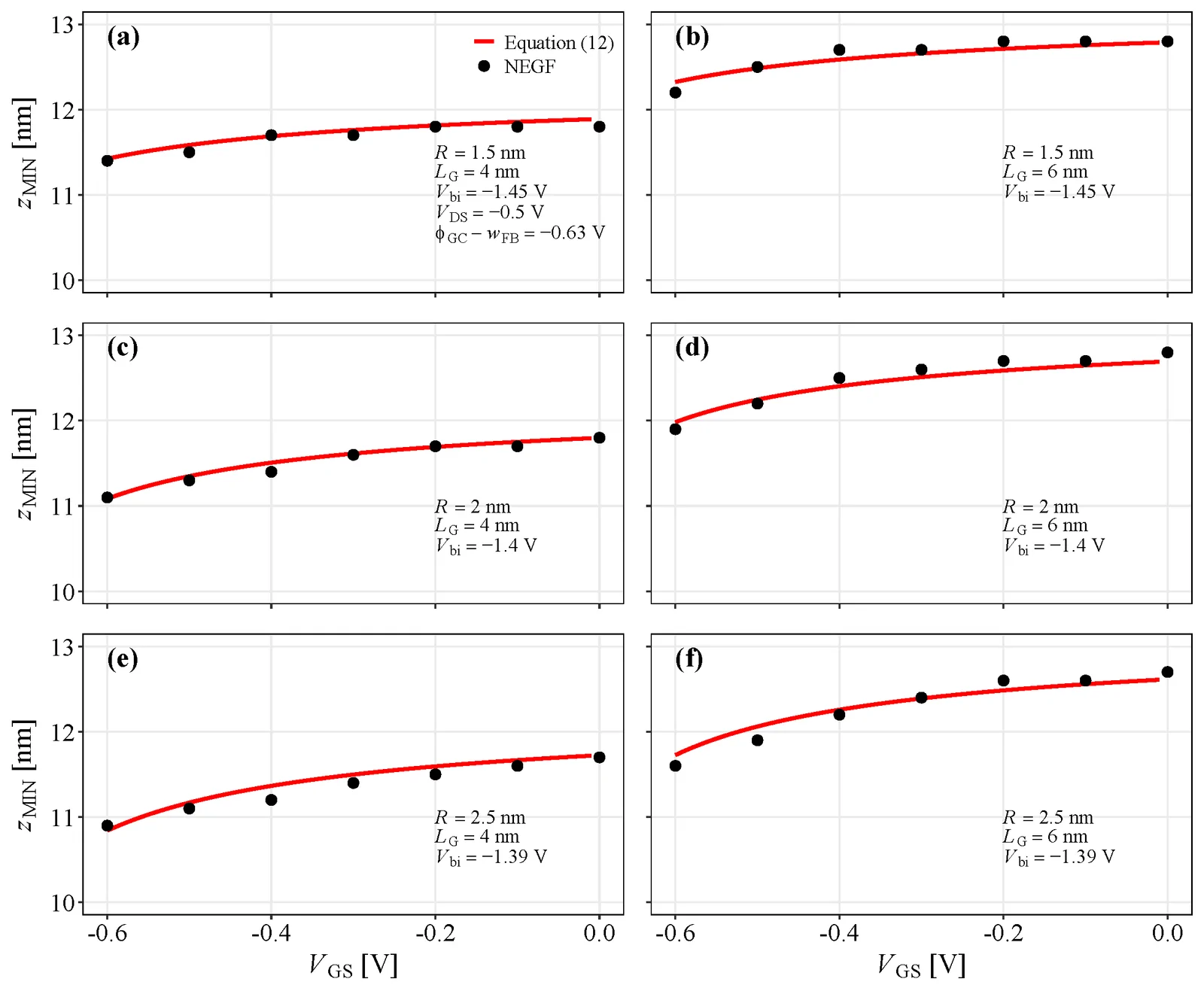
Comparison of the barrier minimum position $z_{\mathrm{MIN}}$ as a function of gate voltage obtained from the NEGF simulations (symbols) and the proposed compact model using Equation (12) (solid lines). (**a**,**b**) R=1.5 nm; (**c**,**d**) R=2 nm; (**e**,**f**) R=2.5 nm. The left column corresponds to $L_{G}=4$ nm and the right column to $L_{G}=6$ nm. All panels are calculated at $V_{\mathrm{DS}}=-0.5$ V and $\phi_{\mathrm{GC}}-w_{\mathrm{FB}}=-0.63$ V; the built-in potential of each nanowire radius is annotated in the corresponding panel.

**Figure 9:**
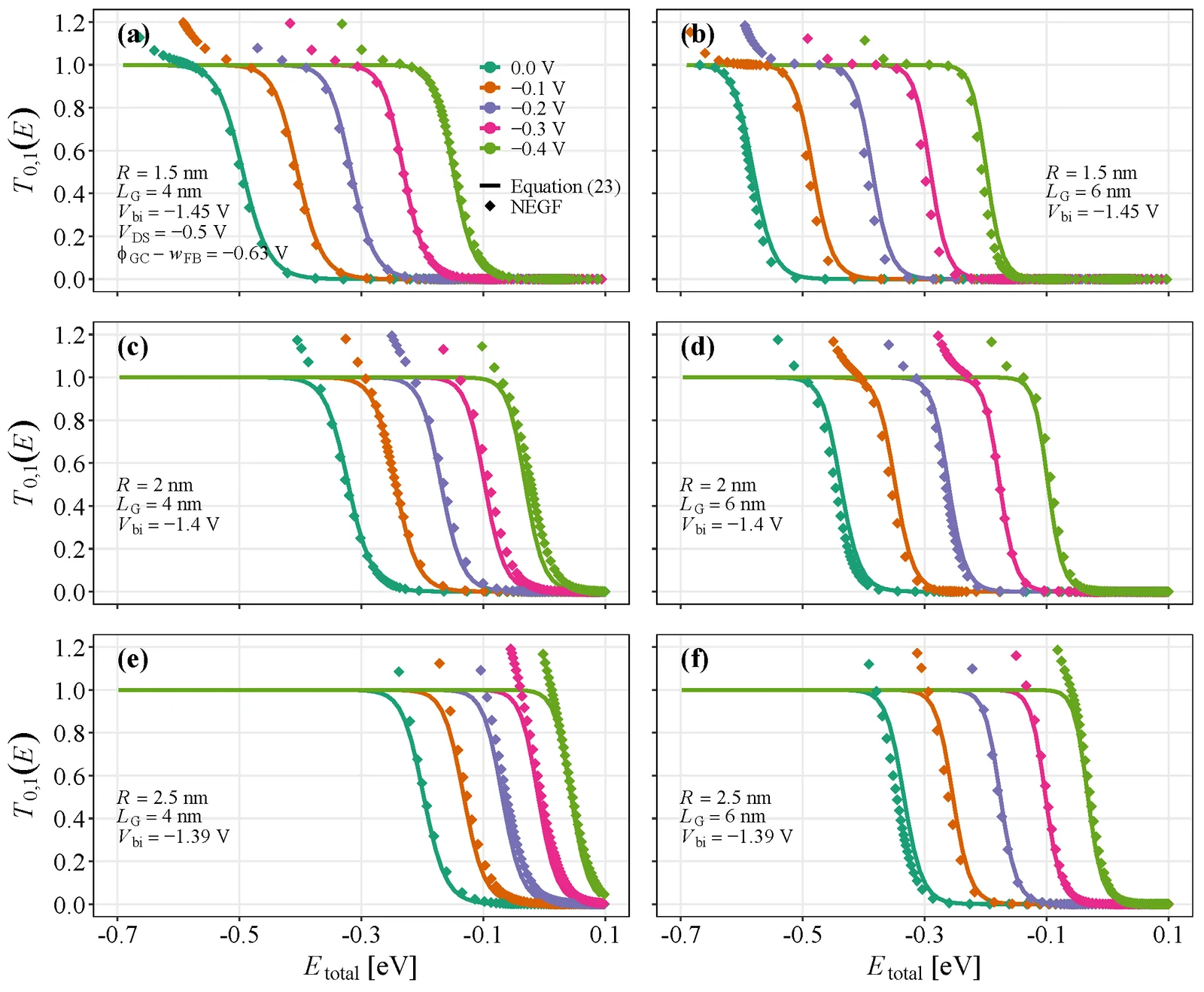
Comparison of the lowest-subband transmission coefficient as a function of total hole energy obtained from the NEGF simulations (symbols) and the proposed analytical compact model using Equation (23) (solid lines). (**a**,**b**) R=1.5 nm; (**c**,**d**) R=2 nm; (**e**,**f**) R=2.5 nm. The left column corresponds to $L_{G}=4$ nm and the right column to $L_{G}=6$ nm. All panels are calculated at $V_{\mathrm{DS}}=-0.5$ V and $\phi_{\mathrm{GC}}-w_{\mathrm{FB}}=-0.63$ V; the built-in potential of each nanowire radius is annotated in the corresponding panel.

Figure 7 demonstrates the lowest hole subband energy level at the barrier minimum, $E_{0,1,\mathrm{MIN}}$, as a function of the gate voltage, calculated using Equation (6). The NEGF simulation results (symbols) are compared with the proposed compact model results (solid lines). Good agreement is observed over the entire gate-voltage range, indicating that the proposed analytical formulation accurately describes the variation in the subband energy level at the barrier minimum in ultra-short-channel p-type nanowire GAA MOSFETs. Figure 8 compares the gate-voltage dependence of the barrier minimum position $z_{\mathrm{MIN}}$. The NEGF simulation results (symbols) are compared with the compact-model results (solid lines) calculated using Equation (12). Good agreement between the two approaches is observed over the investigated bias range. Figure 9 compares the transmission coefficient as a function of the total hole energy $E_{\mathrm{total}}$. The transmission coefficient calculated by the proposed compact model using Equation (23) (solid lines) is compared with the NEGF results (symbols). Good agreement is observed over the entire energy range for gate voltages from 0 to −0.4 V with a step of −0.1 V, where the curves shift from left to right as the gate voltage decreases. It should be noted that the NEGF transmission values can exceed unity because the NEGF simulations employ a multi-subband transport framework, where the total transmission is obtained by summing the contributions from multiple subbands. In contrast, the proposed compact model considers only the lowest subband ($n_{\varphi }=0$, $n_{r}=1$) and therefore gives a single-subband transmission coefficient with a maximum value of unity. Since the lowest subband dominates hole transport under the investigated conditions, the single-subband approximation provides a reasonable description of the transmission characteristics. The good agreement between the compact model and NEGF results confirms the applicability of the proposed transmission coefficient formulation to ultra-short-channel p-type nanowire GAA MOSFETs.

**Figure 10:**
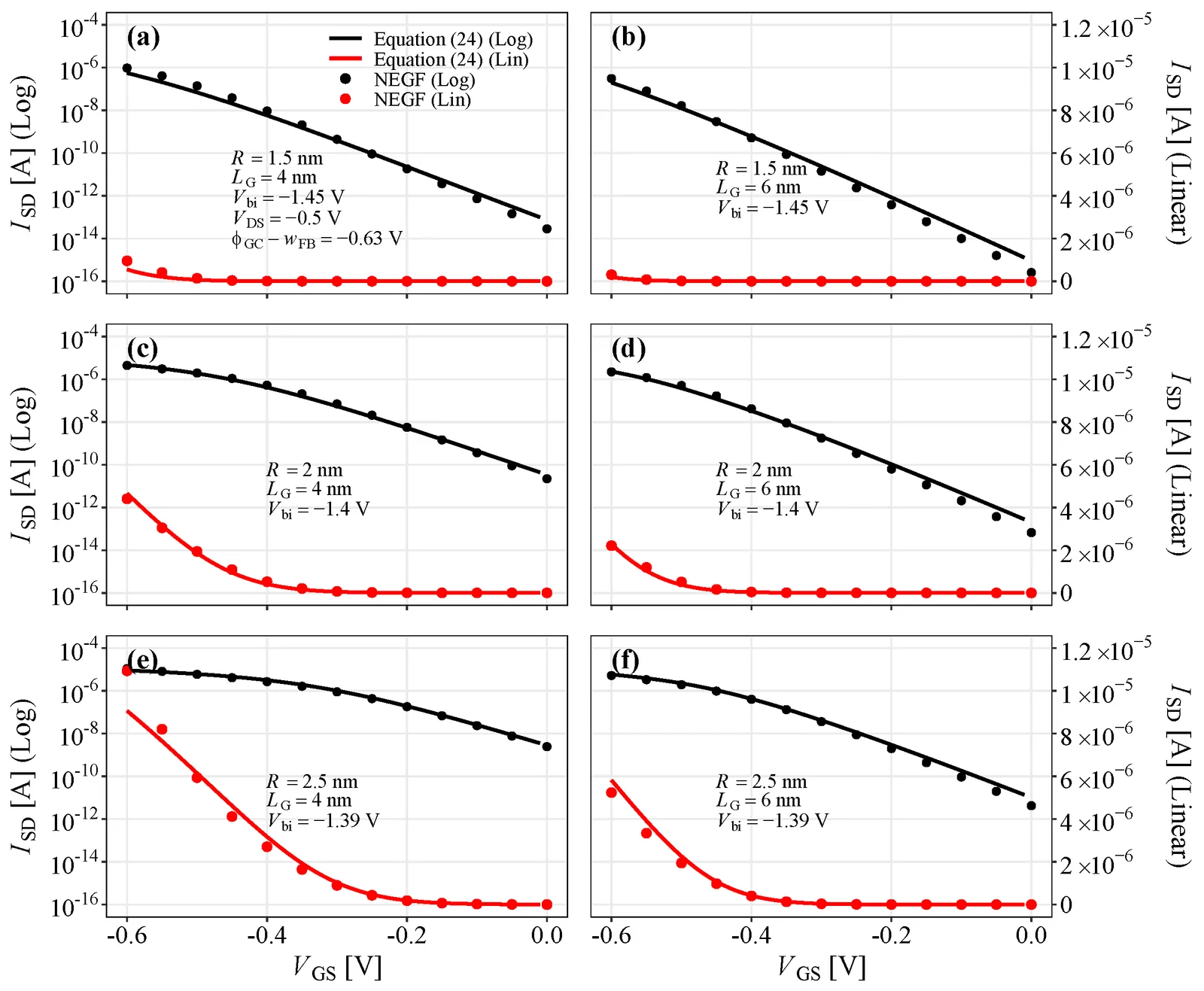
Comparison of the drain current characteristics obtained from the compact model using Equation (24) (solid lines) and NEGF simulations (symbols) over the entire operating range. In each panel the left ordinate is logarithmic and the right ordinate is linear. (**a**,**b**) R=1.5 nm; (**c**,**d**) R=2 nm; (**e**,**f**) R=2.5 nm. The left column corresponds to $L_{G}=4$ nm and the right column to $L_{G}=6$ nm. All panels are calculated at $V_{\mathrm{DS}}=-0.5$ V and $\phi_{\mathrm{GC}}-w_{\mathrm{FB}}=-0.63$ V; the built-in potential of each nanowire radius is annotated in the corresponding panel.

Figure 10 shows the drain current versus gate-voltage characteristics calculated from Equation (24) for various channel lengths and nanowire radii. The compact model results (solid lines) are compared with the corresponding NEGF simulation results (symbols). Reasonable agreement is achieved over the investigated operating regime. In particular, the proposed model captures the increase in subthreshold current caused by SDT while maintaining accurate on-state current prediction for most investigated devices. However, as shown in Figure 10e, a noticeable deviation from the NEGF results is observed in the strong-inversion region. This discrepancy originates from the single-subband approximation adopted in the proposed model, where only the lowest subband is considered, whereas the NEGF simulations include contributions from multiple transport subbands. In the strong-inversion region, higher transport subbands approach the source Fermi level and introduce additional conduction channels, leading to an increase in drain current that is not captured by the single-subband approximation. The deviation is most pronounced for the device with the largest $R/L_{G}$ ratio [27,44], where weakened radial confinement reduces the intersubband energy spacing, and degraded gate electrostatic control further increases the occupation of higher subbands. A multi-subband extension of the present framework will be considered in future work to address this limitation.

**Figure 11:**
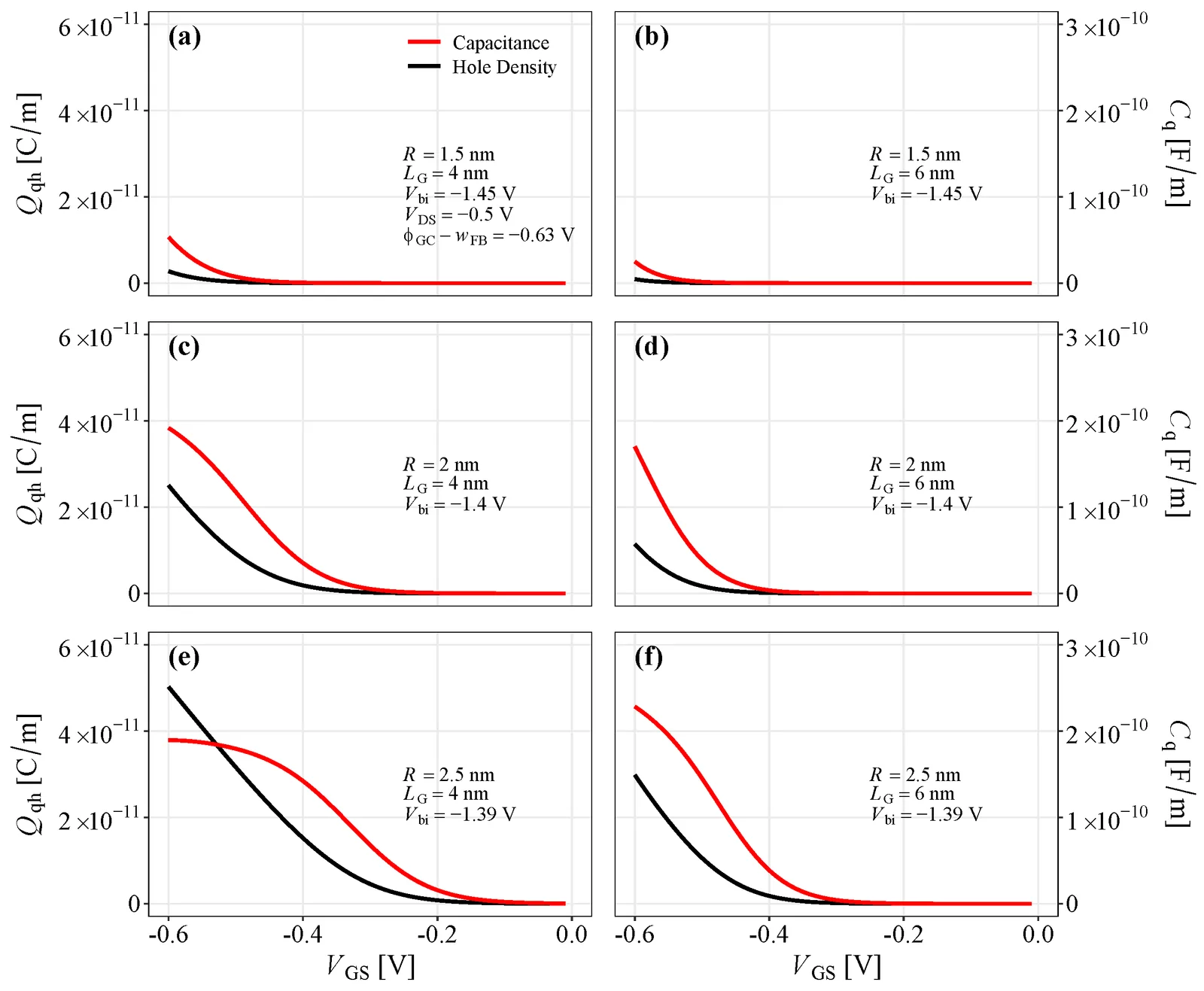
Gate-voltage dependence of the one-dimensional hole charge line density and gate capacitance per unit length, calculated using Equations (25) and (26), respectively. The hole charge line density (left ordinate) is shown by the black curves and the gate capacitance (right ordinate) by the red curves. (**a**,**b**) R=1.5 nm; (**c**,**d**) R=2 nm; (**e**,**f**) R=2.5 nm. The left column corresponds to $L_{G}=4$ nm and the right column to $L_{G}=6$ nm. All panels are calculated at $V_{\mathrm{DS}}=-0.5$ V and $\phi_{\mathrm{GC}}-w_{\mathrm{FB}}=-0.63$ V; the built-in potential of each nanowire radius is annotated in the corresponding panel.

Figure 11 shows the one-dimensional hole charge line density and gate capacitance per unit length as functions of gate voltage, obtained from Equations (25) and (26), respectively. The black and red curves represent the hole charge line density and gate capacitance, respectively. The hole charge line density describes the variation in the channel charge with gate bias, while the gate capacitance characterizes the corresponding differential charge response. As the gate voltage becomes more negative, hole accumulation in the channel increases, leading to an increase in the hole charge line density. Meanwhile, the gate capacitance changes with gate bias due to the nonlinear dependence of the channel charge on the gate voltage. It should be noted that Equation (25) describes the flux-injected charge from the single transport subband considered in the proposed model, consistent with the current formulation. Therefore, the calculated hole charge line density and the derived gate capacitance are not expected to be identical to those obtained from NEGF simulations, where the total hole density includes contributions from higher subbands, source/drain reservoir states, and evanescent states in the barrier.

**Figure 12:**
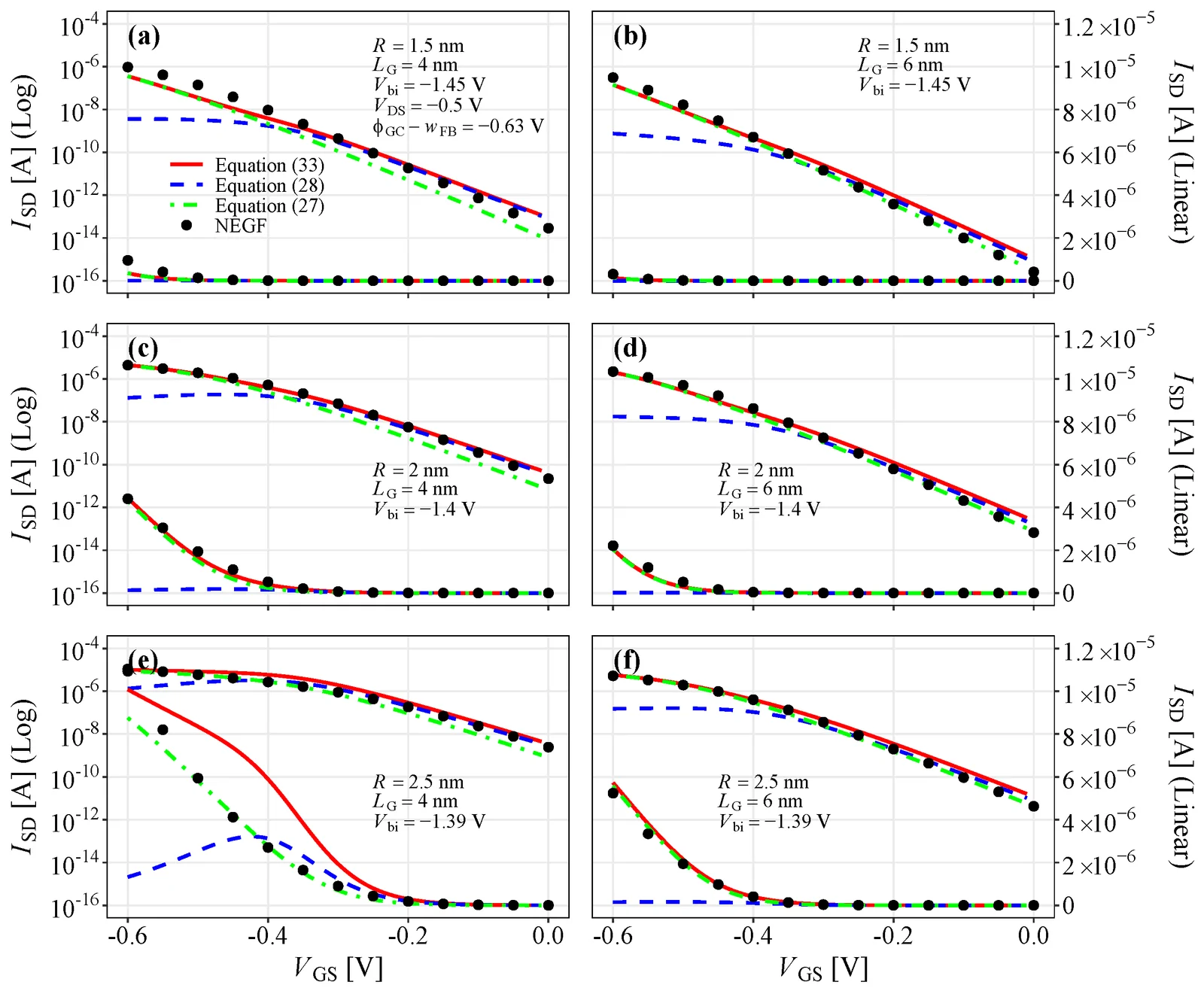
Comparison of the drain current characteristics between the proposed compact model and NEGF simulations (symbols). The thermionic current, tunneling current, and total drain current components calculated from the compact model using Equations (27), (28) and (33) are represented by dashed green, dashed blue, and solid red curves, respectively. In each panel the left ordinate is logarithmic and the right ordinate is linear. (**a**,**b**) R=1.5 nm; (**c**,**d**) R=2 nm; (**e**,**f**) R=2.5 nm. The left column corresponds to $L_{G}=4$ nm and the right column to $L_{G}=6$ nm. All panels are calculated at $V_{\mathrm{DS}}=-0.5$ V and $\phi_{\mathrm{GC}}-w_{\mathrm{FB}}=-0.63$ V; the built-in potential of each nanowire radius is annotated in the corresponding panel.

Figure 12 compares the drain current versus gate-voltage characteristics calculated by the proposed analytical compact model with those obtained from NEGF simulations for various channel lengths and nanowire radii. The thermionic current (dashed green line), tunneling current (dashed blue line), and total current (solid red line) of the analytical model are calculated using Equations (27), (28), and (33), respectively, while the NEGF results are used as the benchmark total drain current. For most investigated devices, the tunneling current is much smaller than the thermionic current in the strong-inversion regime ($V_{\mathrm{GS}}<-0.4$ V), whereas SDT dominates the total current in the subthreshold region and determines the subthreshold leakage level. As shown in Figure 12e (R=2.5 nm, $L_{G}=4$ nm), the tunneling current remains comparable to the thermionic current even in the strong-inversion regime. This deviation is mainly attributed to the overestimation of the tunneling current by the WKB-based formulation in Equation (22), which may produce transmission coefficients exceeding unity, as well as the reduced accuracy of the Boltzmann approximation when the barrier minimum energy approaches the source Fermi level. Nevertheless, the total drain current calculated by the analytical compact model shows reasonable agreement with the NEGF results for most investigated devices, indicating the applicability of the proposed analytical current formulation.

**Figure 13:**
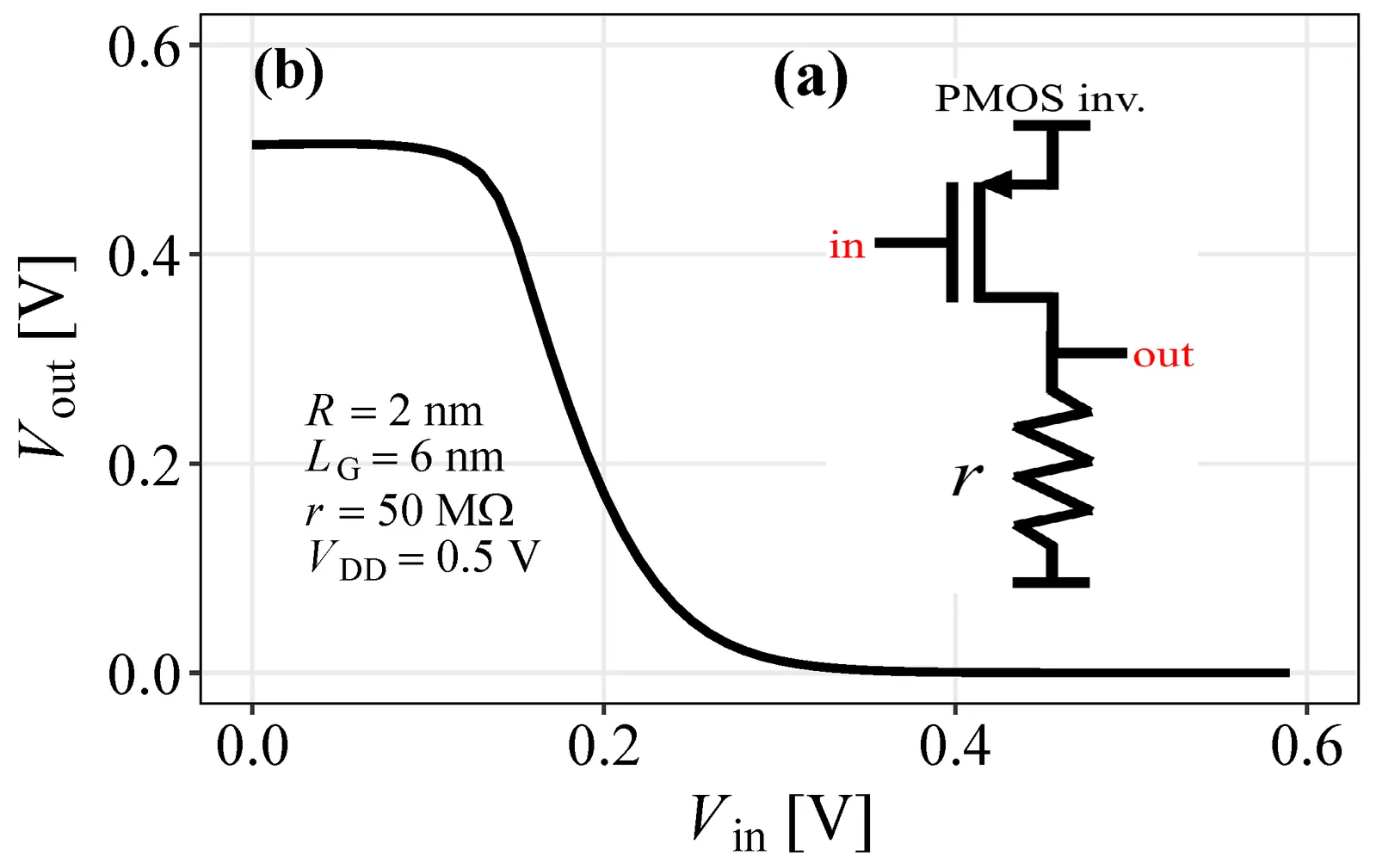
(**a**) PMOS inverter schematic; and (**b**) $V_{\mathrm{OUT}}$–$V_{\mathrm{IN}}$ transfer characteristics obtained from SPICE simulations using the proposed compact model.

Figure 13a shows the resistor-loaded inverter circuit used for SPICE verification. The proposed compact model including the SDT effect is implemented in Verilog-A and simulated in SPICE. Figure 13b presents the simulated voltage transfer characteristics ($V_{\mathrm{OUT}}$ versus $V_{\mathrm{IN}}$) obtained from the DC sweep analysis. The supply voltage is set to $V_{\mathrm{DD}}=0.5$ V [1]. The results demonstrate that the proposed compact model is compatible with SPICE and can reproduce the static transfer characteristics of p-type MOSFET inverter circuits. The current Verilog-A implementation supports DC analyses, including voltage transfer characteristics and operating point simulations.

## 5. Conclusions

This paper presents an analytical compact DC current model together with a numerical gate capacitance model for p-type cylindrical GAA nanowire MOSFETs. The proposed models are developed within the Landauer transport framework, incorporating SDT and quantum statistical charge analysis. The current model is validated against NEGF simulations over a wide range of channel lengths, nanowire radii, and operating biases, showing good agreement in the ballistic limit and capturing the key transport characteristics of ultra-short-channel p-type nanowire GAA MOSFETs.

The model parameters are separated into physical parameters obtained or calibrated from the NEGF simulations and a single set of global empirical fitting parameters. The global empirical fitting parameters are extracted once and applied unchanged across the investigated device geometries and bias conditions, demonstrating their consistency and transferability. The physical parameters obtained or calibrated from the NEGF simulations, including the bandgap, built-in potential, and effective source/drain extension lengths, are fixed once determined and are not re-adjusted for subsequent gate bias conditions. Although quantitative deviations are observed under certain conditions due to the single-subband approximation, the proposed model still provides a reasonable description of the main transport behavior and electrical characteristics. Moreover, the proposed analytical framework can be extended to nanowire transistors with different confinement structures.

It should be noted that the NEGF simulations used in this work assume fully ballistic transport, corresponding to a channel backscattering coefficient of $R_{\mathrm{ref}}=0$. Therefore, the validation presented in this work is limited to the ballistic regime. The channel backscattering coefficient is retained in the Landauer transmission formulation, allowing the framework to be extended to quasi-ballistic transport by introducing a nonzero $R_{\mathrm{ref}}$; however, the accuracy of the model in the quasi-ballistic regime has not been validated in the present work.

Future work will focus on improving the quantitative accuracy by incorporating additional physical effects, including multi-subband transport, valence-band anisotropy for different nanowire orientations, quantum reflection, and parasitic resistances. Validation of $R_{\mathrm{ref}}$ against scattering-inclusive quantum-transport simulations will also be conducted to establish the accuracy of the framework in the quasi-ballistic regime. The robustness of the global parameter set will also be evaluated under temperature variation and geometric scaling. In addition, the Verilog-A implementation will be further developed and benchmarked using standard compact-model validation procedures, including the Gummel symmetry test. Charge-based AC and noise models will be developed to enable accurate small-signal, transient, and noise simulations for future nanoscale CMOS circuit applications.

## Figures and Tables

**Table 1:** Global empirical fitting parameters of the proposed model ^1^.

Fitting Parameters	Value	Equation
$\alpha_{\beta }$	1.3	(4)
$\alpha_{u1}$	0.19	(7)
$\alpha_{u2}$	25,000	(7)
$\alpha_{z}$	0.4	(12)
$\alpha_{T}$	1.2	(23)
$\alpha_{\mathrm{fit}}$	22	(32)
$\beta_{\mathrm{fit}}$	0.37	(32)

^1^ These parameters have no direct physical meaning. They constitute a single global set, determined once and used unchanged for all device geometries and bias conditions.

**Table 2:** Device and material parameters obtained or calibrated from the NEGF simulations ^1^.

Parameters	Value	Unit
$V_{\mathrm{bi}}$	−1.45,−1.4,−1.39 ^2^	V
$m_{\mathrm{hh}}^{r}$	$0.49m_{0}$	kg
$m_{\mathrm{hh}}^{z}$	$0.49m_{0}$	kg
$E_{G}$	1.08	eV
$\phi_{\mathrm{GC}}-w_{\mathrm{FB}}$	−0.63	V
$V_{\mathrm{DS}}$	−0.5	V
$\Delta L_{S}$	1.0	nm
$\Delta L_{D}$	1.8,2	nm

^1^ These parameters are not global empirical fitting parameters. The corresponding extraction or calibration procedures are described in Section 4. ^2^ $V_{\mathrm{bi}}$ is referenced to the source Fermi level under the sign convention defined in Section 2.2 and is therefore negative for the p^+^/intrinsic source junction. Its value depends on the nanowire radius through the confinement-induced change in the effective bandgap.

## Data Availability

The data presented in this study are openly available in GitHub at (https://github.com/hecheng618/TPGAA7nm; accessed on 16 August 2026).
